# Influence of the *GSTP1* rs1695 Polymorphism on Mercury Levels and Memory Performance in the Suruí Indigenous from the Brazilian Amazon

**DOI:** 10.3390/ijerph23060793

**Published:** 2026-06-12

**Authors:** Mayara Calixto da Silva, Paulo Cesar Basta, Bruna Duarte Pinto, Daniel Escorsim Machado, Felipe Oliveira Pessoa-Silva, Rogério Adas Ayres de Oliveira, Ana Claudia Santiago de Vasconcellos, Jamila Alessandra Perini

**Affiliations:** 1 Research Laboratory of Pharmaceutical Sciences (LAPESF), Pharmacy Department (DepFarm), Rio de Janeiro State University (UERJ), Rio de Janeiro 23070-200, Brazil; felipefacfarmacia@gmail.com; 2 Program of Post-Graduation in Epidemiology in Public Health, National School of Public Health (ENSP), Oswald Cruz Foundation (Fiocruz), Rio de Janeiro 21040-900, Brazil; paulobasta@gmail.com; 3 Department of Endemic Diseases Samuel Pessoa, National School of Public Health (ENSP), Oswald Cruz Foundation (Fiocruz), Rio de Janeiro 21041-210, Brazil; 4 Department of Neurology, Hospital das Clínicas, Faculty of Medicine, University of São Paulo (USP), São Paulo 05403-000, Brazil; brunaduartencl@gmail.com; 5 Clinical Analysis Research Laboratory (LAPAC), Pharmacy Department (DepFarm), Rio de Janeiro State University (UERJ), Rio de Janeiro 23070-200, Brazil; danielescorsim@yahoo.com.br; 6 Faculty of Medicine, University of São Paulo (USP), São Paulo 01246-903, Brazil; roger.adas.doc@gmail.com; 7 Laboratory of Professional Education in Health Surveillance, Polytechnic School of Health Joaquim Venâcio (EPSJV), Oswald Cruz Foundation (Fiocruz), Rio de Janeiro 21040-900, Brazil; anacsvasconcellos@gmail.com; 8 Postgraduation Program in Environmental Science and Technology, Rio de Janeiro State University (UERJ), Rio de Janeiro 23070-200, Brazil

**Keywords:** mercury exposure, methylmercury, Indigenous people, neurotoxicity, *GSTP1*, genetics, Brazilian Amazon

## Abstract

**Highlights:**

**Public health relevance—How does this work relate to a public health issue?**

- Mercury exposure from artisanal and small-scale gold mining remains a major public health concern in the Amazon, affecting Indigenous populations with high fish consumption and limited access to health services.
- This study investigates mercury exposure, neurological outcomes, and genetic susceptibility in Paiter-Suruí Indigenous people, addressing an important environmental health issue in vulnerable populations.

**Public health significance—Why is this work of significance to public health?**

- Our findings demonstrate that higher mercury levels are associated with neurological alterations, including impaired memory and reduced muscle strength, highlighting the ongoing health impact of mercury exposure in Amazonian Indigenous communities.
- The *GSTP1* rs1695 polymorphism influenced mercury levels and modified the relationship between mercury exposure and cognitive outcomes, suggesting that genetic variability contributes to individual susceptibility to mercury neurotoxicity.

**Public health implications—What are the key implications or messages for practitioners, policy makers and/or researchers in public health?**

- Identifying genetic factors associated with mercury susceptibility may help prioritize high-risk groups for monitoring, prevention strategies, and targeted public health interventions in exposed populations.
- These findings reinforce the need for stronger environmental policies to reduce mercury contamination from gold mining and support health surveillance programs in Indigenous territories.

**Abstract:**

Mercury (Hg) is a major neurotoxicant and public health concern and gold mining is a significant source of Hg contamination in the Amazon. There, Indigenous peoples are vulnerable to this exposure. Individual susceptibility influences both internal mercury levels and related clinical outcomes. In this context, the *GSTP1* gene stands out due to its role in detoxification of xenobiotics. The objectives were to assess the associations between: (1) Hg levels and neurotoxicity signs; (2) the *GSTP1* rs1695 polymorphism and Hg levels; and (3) whether the *GSTP1* rs1695 polymorphism modifies the effect of mercury on neurotoxicity signs. A cross-sectional study was conducted between April and May 2023, with 113 Paiter-Suruí Indigenous people. Sociodemographic and clinical data were collected using a validated methodology. Hair and oral mucosa cells were collected to assess Hg levels and the *GSTP1* rs1695 polymorphism. Hg levels ranged from 0.1 μg/g to 6.5 μg/g (median = 1 μg/g, IQR = 1.43). Individuals with impaired memory and muscle strength had significantly higher mercury levels (β = 4.39 and β = 1.24). Carriers of the *GSTP1*AA genotype showed a 0.46-point reduction for each 1 μg/g increase in mean Hg levels, compared to individuals with the *GSTP1*GG genotype (β = −0.46). These results may support public policies by identifying priority groups for intervention based on genetic profiles.

## 1. Introduction

Mercury (Hg) is one of the most toxic elements in the environment and represents a major global public health concern, particularly in its organic form, methylmercury (MeHg) [1]. Artisanal and small-scale gold mining (ASGM), also known as *garimpo*, is the main source of environmental mercury contamination, releasing large amounts of elemental mercury (Hg^0^) into the environment, especially in the Amazon region. Between 1975 and 2002, approximately 3000 tons of mercury were released, mostly in South America [2], and in recent years, ASGM activity has increased by more than 90% in the Brazilian Amazon [3].

When Hg^0^ reaches aquatic environments, it is methylated by microorganisms, forming MeHg, which bioaccumulates in fish. Once in the human body, MeHg is rapidly absorbed into the bloodstream and distributed to various organs, with the central nervous system (CNS) being its primary target. It then accumulates in the brain, leading to signs and symptoms of intoxication such as impairments in gait, coordination, cognition, visual function, and somatosensory perception in adults. These irreversible conditions significantly impair the ability of Indigenous populations to engage in work activities and, consequently, to secure livelihoods and generate income [4,5,6,7]. Amazonian Indigenous peoples are among the world’s largest fish consumers and are therefore particularly vulnerable to chronic MeHg exposure [5,7,8]. On the other hand, they are also known to frequently consume Brazil nuts, which are rich in selenium—a powerful antioxidant mineral believed to sequester mercury and reduce its biological availability [9]. In this context, studies focused on understanding the consequences of mercury contamination in Indigenous populations, as well as individual susceptibility, remain limited [10].

Several studies have shown that individual genetic variability, including single-nucleotide polymorphisms (SNPs), may influence the toxicokinetics of MeHg, affecting both internal mercury levels and clinical outcomes [11,12,13,14,15,16,17,18]. One important gene involved in MeHg metabolism is *GSTP1*, which encodes the enzyme glutathione S-transferase pi 1. This enzyme catalyzes the conjugation of MeHg with glutathione (GSH), a key step for mercury detoxification and biliary excretion via the ABC transporter system [19,20,21]. Of the known polymorphisms in this gene, rs1695 SNP (313 A>G) has been widely studied due to its impact on enzyme activity and its prevalence in various populations. This SNP leads to an amino acid substitution at position 105 (Ile105Val), which alters the geometry of the enzyme’s substrate binding site, reducing substrate affinity by approximately threefold and potentially impairing MeHg detoxification [22,23,24,25,26].

Considering the growing concern regarding MeHg exposure and the potential influence of genetic factors, this study aimed to (1) evaluate the association between mercury exposure levels and the prevalence of neurological impairments; (2) assess the association between the *GSTP1* rs1695 polymorphism and mercury exposure levels; and (3) investigate whether the *GSTP1* rs1695 polymorphism modifies the effect of mercury exposure on the signs and symptoms of neurotoxicity among the Paiter-Suruí Indigenous people of the Brazilian Amazon.

## 2. Materials and Methods

### 2.1. Study Design and Ethical Approval

The present study is part of a major project approved by the National Ethics Committee of Human Research (protocol number 65671517.1.0000.5240). A cross-sectional study was conducted between 30 April and 6 May 2023, involving Paiter-Suruí Indigenous individuals residing in the Sete de Setembro Indigenous Land, located in the state of Rondônia, Brazilian Amazon.

### 2.2. Study Area and Population

Before the first contact with the surrounding society, in 1969, the Paiter Suruí population (which in English means “real people, ourselves”) was estimated at around 5000 individuals and inhabited a continuous forest corridor that extended across the states of Rondônia and Mato Grosso, where other Indigenous peoples such as the Gavião, Zoró e Cinta Larga, also lived [27]. However, in 1974, epidemics of influenza, measles, and tuberculosis caused a drastic population decline, leaving only 170 individuals by 1974 [28].

The Sete de Setembro Indigenous Land was officially recognized in 1983 and is located between the states of Rondônia and Mato Grosso, Brazil, covering a territorial area of 248,146.93 hectares. Nowadays, the Paiter Suruí people comprises around 1350 individuals, organized into 25 villages, most of whom live in the municipality of Cacoal, in the southwestern Brazilian Amazon. The interaction with the non-Indigenous world, initiated in 1969, imposes cultural adaptation challenges on the Suruí people, such as dependence on technology, labor relations, and commercial exchanges. However, they still maintain a strong connection to their territory, reinforced by their own strategies of governance, environmental management, and cultural preservation.

### 2.3. Fieldwork and Data Collection

Fieldwork was conducted in Lapetanha Village, from 30 April to 6 May 2023, during which all neighboring villages were also invited to participate. Individuals residing in Lapetanha and five neighboring villages, who agreed to participate were included as a convenience sample (*n* = 178). For the present study, all adults (i.e., individuals aged over 12 years, according to cultural and clinical criteria) who lived inside the Indigenous Land, underwent a neurological evaluation, and provided both hair and oral mucosa samples (further details below) were included (*n* = 113). The sample of children will be included in a forthcoming study.

**Figure 1:**
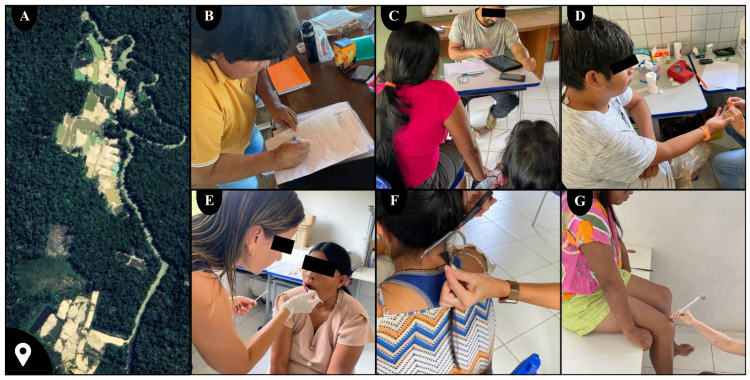
Visual records of the field work adopted methodology. (**A**) Lapetanha Indigenous village; (**B**) signature of the consent form; (**C**) application of the sociodemographic data questionnaire; (**D**) clinical and laboratory assessments; (**E**) oral mucosal cells collection; (**F**) hair sample collection; (**G**) neurological evaluation.

After providing written informed consent (Figure 1B), participants answered a sociodemographic questionnaire administered by the research team during initial home visits and family interviews (Figure 1C).

### 2.4. Clinical and Neurological Assessment

Participants were then referred to a clinical evaluation, during which clinical and laboratory assessments were conducted (Figure 1D), hair and oral mucosa samples were collected (Figure 1E,F), and anthropometric measures were taken. BMI was calculated as weight (kg) divided by the square of height (m^2^). A standardized neurological examination protocol, specifically developed for this research, was carried out by the neurologist and co-author BDP (Figure 1G). Both self-reported symptoms and clinically observed signs were evaluated across three neurological domains: somatosensory, motor/coordination, and cognitive.

The overall cognitive function was assessed through the Brief Cognitive Screening Battery (BCSB) and the semantic verbal fluency test (S-VFT) in the animal category. Cutoff points of <6 in the BCSB and <9 in the S-VFT were used to determine Abnormal/Altered results. Motor function assessment included muscular strength and rigidity of proximal and distal segments of all four limbs, as well as bradykinesia, coordination, deep osteotendinous ankle reflex, toe amyotrophy, static balance and gait. Finally, the somatosensory functions/receptors evaluated were the mechanoreceptors (detection of light touch, vibration, pressure, and texture), nociceptors (detection of pain) and thermoreceptors (detection of temperature), comparing upper versus lower limbs, left side versus right side, as well as proximal versus distal levels. The detailed neurological assessment protocol has been previously published [10].

### 2.5. Biological Samples Collection

Hair samples of approximately 1 cm in diameter were collected from the occipital region of the scalp using stainless-steel dissection scissors to estimate participants’ internal total Hg dose (exposure levels). The samples were then stored in individually identified paper envelopes and sent to the Research Laboratory of Pharmaceutical Sciences (LAPESF) (https://lapesfuerjzo.my.canva.site/ (accessed on 7 April 2026)) of Rio de Janeiro State University (UERJ) in Rio de Janeiro-RJ, for analysis of total mercury levels.

Epithelial cells were collected from the oral mucosa using sterile swabs, to access the genomic DNA of the participants. Then, the swabs were identified and individually stored in microtubes containing 300 µL of a phosphate-buffered solution of 0.01 M (pH 7.45) prepared from a mixture of 0.1 M Na_2_HPO_4_ and NaH_2_PO_4_ buffers (72 mL and 28 mL, respectively), 9 g of NaCl, and distilled water to a final volume of 1 L. Subsequently, the samples (swab stored in the buffer solution) were transported to LAPESF (https://lapesfuerjzo.my.canva.site/), in Rio de Janeiro-RJ, for genetic polymorphism analysis.

### 2.6. Hair Mercury Analysis

Total mercury (THg) from hair samples is known to be a reliable biomarker of MeHg exposure through consumption of contaminated fish, since MeHg is the predominant form of mercury found in hair THg. Since hair grows approximately 1–1.5 cm per month, the analysis of 1 cm of proximal hair provides an estimate of exposure levels during the 30 days preceding sample collection [29,30]. THg analysis in hair samples was performed using the RA-915M mercury analyzer coupled with the PYRO-915+ pyrolysis unit (Lumex Instruments, Mission, BC, Canada). The PYRO-915+ thermal unit promotes the reduction in Hg(II) to its atomic state through thermal decomposition (pyrolysis) of the sample. Subsequently, mercury atoms are transported by air flow to the analytical cell of the RA-915M analyzer. The mercury mass concentration is then calculated based on the integration of the analytical signal (peak) and a previously established calibration curve (peak area vs. mercury mass), using the Zeeman-effect background correction atomic absorption spectrometry method (Zeeman AAS).

Approximately 1–1.5 cm from the proximal end of each hair sample was cut and then ground using stainless steel scissors. Then, three replicates (≥6.0 mg each) were weighed using the quartz boats that were inserted into the PYRO-915+ thermal unit. The results correspond to the arithmetic mean of the three measurements, provided that the coefficient of variation (CV) was below 15%. If the CV exceeded 15%, a fourth measurement was performed, and the most discrepant value among the four was excluded. To ensure the reliability of the results, calibrations were performed on the day prior to the start of the analyses. Additionally, for every 10 samples analyzed, approximately 10 mg of certified reference material (ERM–DB001, Human Hair, IRMM, Geel, Belgium) was weighed and analyzed directly, following the manufacturer’s instructions. All reference material analyses remained within the acceptable range. Mercury levels above 2.0 µg/g of hair were considered high according to the manual recently published by the Brazilian Ministry of Health in partnership with the Oswaldo Cruz Foundation [31].

### 2.7. DNA Extraction and GSTP1 Polymorphism Genotyping

Genomic DNA extraction was performed using a standardized extraction kit (Qiagen, Hilden, Germany), following the manufacturer’s instructions. Briefly, to release intracellular material, 20 µL of proteinase and 400 µL of lysis buffer was added to a new microtube containing 200 µL of the sample and the mixture was incubated at 56 °C for 20 min. To allow DNA precipitation, the tubes were centrifuged and 400 µL of ethanol was added. Then, 700 µL of the mixture was transferred to a silica column tube, which, after 5 min of centrifugation, retains the DNA. Finally, two washing steps and one elution step of the purified DNA were performed to provide high quality purified DNA.

Genotyping of the *GSTP1* (chr11:67585218) 313 A>G (rs1695) missense variant was performed using the 7500 Real-Time PCR System (Applied Biosystems, Foster City, CA, USA), followed by a TaqMan allelic discrimination assay (C_3237198_20, Applied Biosystems), as previously described [16,17]. Genotypes were determined directly by gene counting.

### 2.8. Variables and Statistical Analysis

The primary exposure and outcome of interest are mercury levels and results of the neurological assessment, respectively. Furthermore, the genetic polymorphism will be evaluated both as an exposure variable for neurological outcomes and as a potential effect modifier of mercury levels on the results of the neurological assessment.

Mean and median values, as well as their respective measures of dispersion, were used to present the continuous variables (age, monthly income and mercury exposure levels) and the Shapiro–Wilk test was applied to assess their normality. Kruskal–Wallis test, followed by the Dunn post hoc test, were used to investigate differences between groups. Spearman’s linear correlation coefficient and linear regression models adjusted for possible confounding factors were used to test the correlation between continuous variables. Absolute (n) and relative frequencies (%) were used to present categorical variables, while the Pearson’s chi-square test or Fisher’s exact test, when necessary, were used to test the differences between groups. The Hardy–Weinberg equilibrium (HWE) for the *GSTP1* 313 A>G polymorphism was calculated by the goodness-of-fit 2 test.

It is known that sex and age are strongly associated with mercury exposure levels; therefore, these variables were considered confounding factors and were used to adjust the mercury levels in the multivariate models. All statistical analyzes were performed using the R Software (R Foundation for Statistical Computing, Vienna, Austria, version 4.2.2) and a significance level of 5% was adopted.

## 3. Results

A total of 113 individuals were included in the study, most of whom were female, with a median age of 32 years. Most participants were overweight, married, and were either attending or had completed secondary education. The predominant monthly income ranged from R$1501 to R$7200, and most individuals consume fish and Brazilian nut at least once a week (Table 1).

Mercury levels from the 113 samples ranged from 0.11 μg/g to 6.5 μg/g in the general population, with a mean of 1.42 μg/g (SD = 1.37) and a median of 1.00 μg/g (IQR = 1.43), showing a non-normal distribution (*p*-value < 0.05, Shapiro–Wilk test). Only 26 individuals had mercury levels higher than 2.0 μg/g.

In the analysis of sociodemographic and clinical characteristics (sex, age, BMI, marital status, education level, monthly income, fish and Brazil nut consumption) according to mercury levels, only age and sex remained statistically significant in the multivariable analysis (Table 2) and, therefore, were included as covariates in the subsequent analyses.

Table 3 presents the linear regression models exploring the association between neurological evaluation outcomes and mercury exposure levels from the 113 individuals assessed. Positive associations were observed between altered memory and muscle strength and mercury levels. Individuals with memory impairments had higher mercury levels (β = 4.39; IC 95%: 2.18–6.60; *p* < 0.001) as did those with reduced muscle strength (β = 1.24; IC 95%: 0.16–2.32; *p* = 0.02), after adjustment for sex, age, and fish consumption. Regarding the self-reported symptoms, no significant associations were found.

The genotypic frequency of the *GSTP1* rs1695 A>G polymorphism was in Hardy–Weinberg equilibrium, with the *GSTP1* G allele frequency at 21.3% in the study population. When assessing the association between the polymorphism and mercury levels, it was observed that individuals with the GG genotype had, on average, 0.95 µg/g more mercury than those with the AA genotype, after adjusting for sex, age and fish consumption (Table 4).

**Figure 2:**
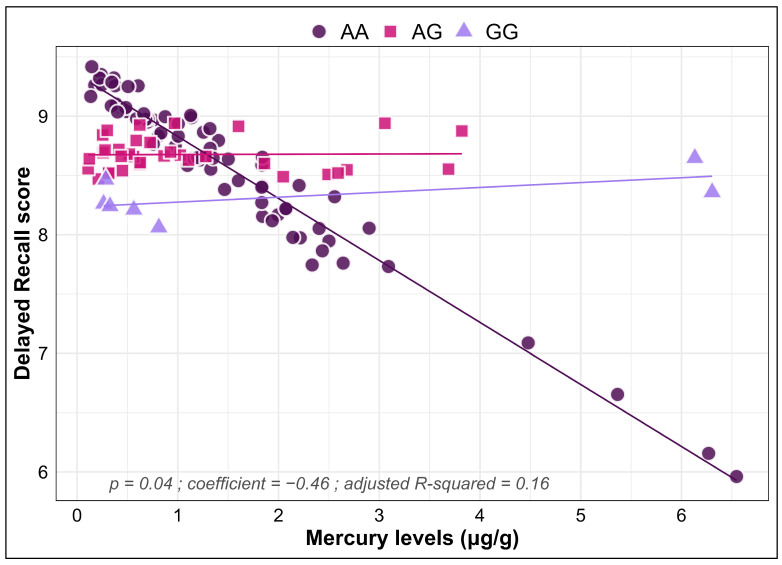
Linear relationship between mercury levels and delayed recall scores modified by the *GSTP1* A>G genotypes. Lines indicate the linear trend between mercury levels and delayed recall scores within each genotype group.

When investigating the linear relationship between neurological evaluation results and mercury levels, a positive association with the delayed recall score was found, in which for every 1 μg/g increase in mean mercury levels, the score on the delayed recall test decreases by an average of 0.24 points (*p*-value = 0.04; Adjusted R^2^ = 0.07), adjusted for sex, age and fish consumption. Furthermore, the relationship between mercury levels and memory differs depending on the presence of the *GSTP1* rs1695 A>G polymorphism (Figure 2). Individuals with the AA genotype show a 0.46-point reduction in the delayed recall score for each 1 μg/g increase in mean mercury levels (*p*-value = 0.04; Adjusted R^2^ = 0.16), whereas no negative effect of mercury on delayed recall was observed in individuals with AG or GG genotypes. The estimated coefficients from the interaction models are presented in Supplementary Table S1.

Finally, considering only the 26 individuals with elevated Hg levels (≥2.0 µg/g), the same associations observed between Hg levels and neurological outcomes and the *GSTP1* polymorphism remained statistically significant (Supplementary Tables S2 and S3). The main characteristics of these individuals are described in Table 5. Most of the individuals had the *GSTP1* rs1695 AA genotype and almost 90% of them presented at least one alteration during the neurological assessment.

## 4. Discussion

This study assessed mercury exposure among the Paiter-Suruí population residing in the Sete de Setembro Indigenous Territory, located in Cacoal, Rondônia. Elevated mercury levels were identified and found to be associated with neurological symptoms commonly linked to mercury-induced neurotoxicity. Furthermore, we observed that the *GSTP1* gene polymorphism may modulate these associations. To the best of our knowledge, this is the first study to investigate these relationships within this Indigenous community.

Indigenous populations in Brazil are currently undergoing a nutritional transition characterized by the replacement of traditional foods with ultra-processed products [32]. This shift is clearly reflected in the present study, as the population under investigation lives in an urbanized context and shows an obesity prevalence 20% higher than the average reported for Indigenous populations [33] and 40% higher than that observed in the general Brazilian population [34]. However, fish consumption remains a cornerstone of the Indigenous diet, especially in more isolated and lower-income communities [32,35].

Thanks to the bioaccumulation potential of mercury over time, age stands out as an important variable for understanding the effects of this exposure, as widely described in the literature [36]. Following the ingestion of a single dose of contaminated fish, it is expected that the human body will eliminate approximately half of the absorbed mercury within 180 days [19]. However, in the context of Indigenous populations where fish consumption is frequent and sustained [32]. mercury tends to accumulate in the body over time, resulting in a positive correlation with age [37,38]. Regarding sex differences, studies have shown that men often exhibit higher mercury levels than women, even when fish consumption is comparable. This is attributable to physiological differences such as muscle mass, organ volume, and intestinal biotransformation rates [17,39,40]. Motor and cognitive disorders are among the most common neuropathological findings associated with mercury intoxication [41]. As expected, in the present study, higher levels of mercury were associated with memory and muscle strength alterations.

The *GSTP1* rs1695 SNP (313 A>G) causes an Ile/Val substitution at position 105 of the GSTP1 enzyme, resulting in reduced enzymatic activity. This alteration is believed to influence mercury levels through decreased mercury conjugation with glutathione, leading to reduced elimination of the metal and consequently increasing the risk of developing neurotoxicity [22,23,24,25,26]. In the present study, the *GSTP1* AA genotype was found to increase the effect of mercury levels on delayed recall scores. This finding is consistent with observations made in another Indigenous population exposed to mercury in the Brazilian Amazon [16]. The observed scores, regardless of the individual’s genotype, are within the range considered normal (≥6 figures identified) [42], yet it is already possible to observe an interaction with the *GSTP1* genotype. These results highlight the importance of identifying individual variability factors, such as genetic polymorphisms, that allow personalized and population-specific strategies to mitigate the impacts of methylmercury exposure. which is a public health concern on the Amazon.

Recently, the Oswaldo Cruz Foundation, in collaboration with the Brazilian Ministry of Health, established that hair mercury levels above 2.0 μg/g are considered alarming, as they are associated with the development of neurotoxicity signs, according to the updated literature [31]. In the present study, however, even mercury levels below this threshold were significantly associated with early signs of neurotoxicity. This finding suggests the importance of early interventions aimed at preventing further increases in mercury exposure—and, consequently, a future increase in neurological impairments within this population. To evaluate the robustness of these findings, a sensitivity analysis was performed only with those 26 individuals that were above the safe limit of 2.0 μg/g. It was observed that mercury levels were still associated with impaired memory and loss of muscle strength, as well as with the *GSTP1* rs1695 GG allele. Furthermore, a high prevalence of neurological alterations was observed, reinforcing that these individuals should be prioritized for active surveillance and follow-up. Therefore, given the neurotoxic effects that mercury can cause, which have been extensively documented in the literature, beginning with the Minamata tragedy, it is important to consider the potential risks associated with its use.

The present study has several strengths that should be highlighted, including access to a relatively isolated population and the importance of including this Indigenous population in a genetic study, as Indigenous peoples are still underrepresented in such studies. Given the variability in the origins of Brazilian Indigenous ethnic groups and their consequent genetic heterogeneity, studying a larger number of ethnic groups increases the potential for extrapolating the findings once a relevant association is identified. In addition, the implementation of this study mobilized a multidisciplinary team composed of pharmacists, biologists, psychologists, and physicians from different specialties to conduct the survey and provide health care services to a vulnerable population with limited access to health care. A few limitations of the present study should also be noted, such as the small representativeness of the sampling method and the impossibility of causal inference for the observed associations, due to the cross-sectional design. Additionally, it is impossible to exclude the influence of non-measured co-exposure neurotoxicants and potential epigenetic mechanisms. Furthermore, the low prevalence of some of the outcomes and the *GSTP1* polymorphism, even when the *GSTP1* SNP is rearranged into the recessive model, may limit the statistical power, as well as the evaluation of multiple neurological outcomes without formal correction for multiple comparisons may have increased the risk of false-positive findings. We believe that these limitations do not compromise the significance of our work; however, they need to be addressed in further studies, particularly of longitudinal designs.

## 5. Conclusions

In conclusion, the present study was able to assess the mercury contamination profile of the Paiter-Suruí Indigenous people, as well as the association between this exposure and the prevalence of neurological impairments. Furthermore, an influence of the *GSTP1* 313 A>G polymorphism on mercury levels was observed, as well as its apparent ability to modify the effect of mercury exposure on the signs and symptoms of neurotoxicity.

## Figures and Tables

**Table 1:** Sociodemographic characteristics of participants, Rondônia, Amazon, Brazil, in 2023.

Characteristics	Overall
	(*n* = 113)
**Sex**	*n* (%)
Female	65 (57.5)
Male	48 (42.5)
**Age (mean ± SD) ^a^**	32.73 (16.3)
**Age group ^a^**	*n* (%)
12–15	12 (10.6)
16–20	17 (15.0)
21–30	36 (31.9)
31–40	17 (15.0)
41 or older	31 (27.4)
**BMI ^b^**	*n* (%)
Malnutrition	2 (1.8)
Normal weight	37 (33.3)
Overweight/Obesity	72 (64.9)
**Marital status ^c^**	*n* (%)
Married	66 (58.9)
Single	39 (34.8)
Widowed	4 (3.6)
Separated	3 (2.7)
**Education level ^d^**	*n* (%)
Fundamental	30 (32.3)
High school	54 (58.1)
Higher education	9 (9.7)
**Income (mean ± SD) ^e^**	2292.48 (1648.6)
**Monthly income ^e^**	*n* (%)
0–1500 BRL	48 (42.5)
1501–7200 BRL	65 (57.5)
**Fish consumption**	*n* (%)
Daily	23 (20.4)
Weekly	71 (62.8)
Monthly	19 (16.8)
**Brazil nut consumption**	*n* (%)
Daily	23 (20.4)
Weekly	71 (62.8)
Monthly	19 (16.8)

^a^ Age in years; ^b^ Missing information from 2 individuals (*n* = 111); ^c^ Missing information from 1 individual (*n* = 112). ^d^ Twenty people have never attended school (*n* = 93). ^e^ Brazilian *reais* (BRL).

**Table 2:** Linear regression models between sociodemographic characteristics and mercury exposure levels in the Sete de Setembro Indigenous Territory, Rondônia, Amazon, Brazil, 2023.

Characteristics	Median (IQR)	*p*-Value (Univariate Analysis)	Multivariable Analysis ^a^			
			Estimate (β)	Standard Error	95% CI	*p*-Value
**Sex**						
Male	1.29 (1.49)	0.13	0.61	0.25	0.12; 1.10	**0.02**
**Age (years)**	**1.00 (1.43)**	**<0.01**	0.05	0.01	0.02; 0.07	**<0.01**
**BMI (Kg/m^2^)**						
Undernutrition	0.45 (0.17)	0.30	−1.14	0.82	−2.77; 0.50	0.17
Overweight/Obesity	1.11 (1.47)	0.74	−0.61	0.31	−1.25; 0.01	0.05
**Marital status**						
Single	**0.62 (0.89)**	**0.01**	0.20	0.83	−1.45; 1.84	0.81
Widower	1.99 (0.47)	0.93	0.07	0.31	−0.55; 0.68	0.83
Separated	0.98 (0.51)	0.35	−0.35	1.14	−2.61; 1.91	0.76
**Education level**						
Primary	**1.11 (1.39)**	**<0.01**	0.38	0.27	−0.16; 0.92	0.17
Higher education	0.84 (0.52)	0.92	−0.52	0.46	−1.43; 0.40	0.27
**Monthly income**						
0–1500 BRL	1.00 (1.43)	0.40	0.27	0.25	−0.23; 0.78	0.29
**Fish consumption ^b^**						
Yes	1.01 (1.45)	0.41	1.0	0.98	−0.91; 2.97	0.29
**Brazil nut consumption**						
Weekly	**1.22 (1.95)**	**0.02**	0.74	0.39	−0.03; 1.50	0.06
Monthly	**1.13 (1.40)**	**0.01**	0.44	0.30	−0.17; 1.04	0.15

^a^ Complete model containing all variables (sex, age, BMI, marital status, education level, monthly income, fish and Brazil nut consumption); ^b^ Fish consumption at least once a month. Bold indicates statistically significant results.

**Table 3:** Linear regression models between neurological evaluation outcomes and mercury exposure levels, Sete de Setembro Indigenous Territory, Rondônia, Amazon, Brazil, 2023.

Signs	*n* (%)	Multivariable Analysis ^a^			
	(*n* = 113)	Estimate (β)	Standard Error	95% CI	*p*-Value
**Cognitive function**					
Verbal fluency	26 (24.1)	0.14	0.27	−0.39; 0.67	0.59
Memory	1 (0.9)	**4.39**	**1.11**	**2.18; 6.60**	**<0.01**
Stick test	19 (17.8)	−0.25	0.35	−0.95; 0.44	0.47
Cognition	46 (42.6)	0.23	0.24	−0.24; 0.71	0.33
**Motor function**					
Muscle strength	5 (4.7)	**1.24**	**0.54**	**0.16; 2.32**	**0.02**
Muscle rigidity	3 (2.8)	0.09	0.73	−1.35; 1.55	0.89
Toe amyotrophy	1 (0.9)	−1.44	1.20	−3.82; 0.94	0.23
Bradykinesia	3 (3.1)	0.46	0.70	−0.93; 1.86	0.51
Deep tendon reflexes	25 (24.0)	−0.01	0.31	−0.62; 0.59	0.95
Gait	1 (1.0)	−0.53	1.21	−2.94; 1.88	0.66
**Somatosensory function**					
Tactile sensitivity	5 (4.9)	−0.28	0.57	−1.41; 0.84	0.62
Deep sensitivity	15 (13.8)	−0.06	0.40	−0.85; 0.72	0.87
Thermal sensitivity	19 (17.4)	−0.34	0.30	−0.93; 0.26	0.27
Nociception	17 (15.6)	−0.50	0.31	−1.12; 0.12	0.11
Visual field	2 (1.8)	−0.95	0.84	−2.61; 0.71	0.26

^a^ Adjusted for sex, age and fish consumption. Bold indicates statistically significant results.

**Table 4:** Linear regression models between the *GSTP1* SNP and mercury exposure levels, Sete de Setembro Indigenous Territory, Rondônia, Amazon, Brazil, 2023.

*GSTP1* rs1695 A>G	Median (IQR)	Multivariable Analysis			
		Estimate (β)	Standard Error	95% CI	*p*-Value ^a^
AA	1.21 (1.36)				
AG	0.62 (1.18)	−0.20	0.24	−0.68; 0.28	0.41
GG	0.56 (3.17)	**0.94**	**0.46**	**0.03; 1.84**	**0.04**
AA + AG	1.02 (1.39)				
GG	0.56 (3.17)	**1.00**	**0.45**	**0.11; 1.89**	**0.03**
A	1.13 (1.43)				
G	0.62 (1.35)	0.17	0.18	−0.19; 0.54	0.36

^a^ Adjusted for sex, age and fish consumption. Bold indicates statistically significant results.

**Table 5:** Sociodemographic characteristics of the 26 participants with Hg levels ≥ 2.0 µg/g, Rondônia, Amazon, Brazil, in 2023.

Characteristics	Overall
	(*n* = 26)
**Sex**	***n* (%)**
Female	14 (53.8)
Male	12 (46.2)
**Age (mean ± SD) ^a^**	48.85 (16.42)
**Age group ^a^**	***n* (%)**
16–20	2 (7.7)
21–30	3 (11.5)
31–40	3 (11.5)
41 or older	18 (69.2)
**BMI ^b^**	***n* (%)**
Normal weight	8 (32.0)
Overweight/Obesity	17 (68.0)
**Marital status**	***n* (%)**
Married	21 (80.8)
Single	3 (11.5)
Widowed	2 (7.7)
**Education level ^c^**	***n* (%)**
Fundamental	7 (58.3)
High school	5 (41.7)
**Income (mean ± SD) ^d^**	2207.7 (1776.5)
**Monthly income ^d^**	***n* (%)**
0–1500 BRL	12 (46.2)
1501–7200 BRL	14 (53.8)
**Fish consumption ^e^**	***n* (%)**
No	0 (0.0)
Yes	26 (100.0)
**Fish consumption**	***n* (%)**
Daily	2 (7.7)
Weekly	7 (26.9)
Monthly	17 (65.4)
**Brazil nut consumption**	***n* (%)**
Daily	23 (20.4)
Weekly	71 (62.8)
Monthly	19 (16.8)
***GSTP1* 313 A>G**	***n* (%)**
AA	17 (65.4)
AG	7 (26.9)
GG	2 (7.7)
**Neurological findings ^f^**	***n* (%)**
None	3 (11.5)
1 to 3	13 (50.0)
4 or more	10 (38.5)

^a^ Age in years; ^b^ Missing information from 1 individual (*n* = 25); ^c^ fourteen people have never attended school (*n* = 12). ^d^ Brazilian *reais* (BRL); ^e^ fish consumption at least once a month; ^f^ any alteration found during the neurological evaluation (cognitive, motor or somatosensory).

## Data Availability

Data are contained within the article and Supplementary Materials.

## Supplementary Materials

The following supporting information can be downloaded at https://www.mdpi.com/article/10.3390/ijerph23060793/s1. Table S1: Linear regression models for the interactions between the *GSTP1* rs1695 A>G polymorphism and Hg Levels, Sete de Setembro Indigenous Territory, Rondônia, Amazon, Brazil, 2023; Table S2: Linear regression models between neurological evaluation outcomes and mercury exposure levels among the 26 participants with Hg levels ≥ 2.0 µg/g, Sete de Setembro Indigenous Territory, Rondônia, Amazon, Brazil, 2023; Table S3: Linear regression models between the *GSTP1* SNP and mercury exposure levels among the 26 participants with Hg levels ≥ 2.0 µg/g, Sete de Setembro Indigenous Territory, Rondônia, Amazon, Brazil, 2023.
