# Supplementary material for: Influence of the GSTP1 rs1695 Polymorphism on Mercury Levels and Memory Performance in the Suruí Indigenous from the Brazilian Amazon
Source: Int J Environ Res Public Health. 2026 Jun 12;23(6):793. doi: 10.3390/ijerph23060793 (PMC13299861; doi:10.3390/ijerph23060793)
Supplement: Supplementary file 1 [file ijerph-23-00793-s001.zip › Supplementary Table S1.pdf]

Supplementary Table S1. Linear regression models for the interactions between the *GSTP1* rs1695 A>G polymorphism and Hg Levels, Sete de Setembro Indigenous Territory, Rondônia, Amazon, Brazil, 2023.

***GSTP1* rs1695 A>G**	**Overall (n= 113)**	**Multivariable analysis[a]**			
		**Estimate (β)**	**Standard Error**	**95% CI**	**P-value**
AA[b]	72 (63.7)	**-0.47**	**0.19**	**-0.85 : -0.09**	**0.01**
AG	34 (30.1)	**0.51**	**0.25**	**0.01 : 1.01**	**0.04**
GG	7 (6.2)	**0.50**	**0.24**	**0.02 : 0.98**	**0.04**
AA	72 (63.7)				
AG + GG	41 (36.3)	**0.47**	**0.19**	**0.1 : 0.85**	**0.01**
AA + AG	106 (93.8)				
GG	7 (6.2)	0.37	0.23	-0.09 : 0.83	0.11

[a]Adjusted for sex, age and fish consumption. [b]Using the GG genotype as reference.
