# Supplementary material for: Influence of the GSTP1 rs1695 Polymorphism on Mercury Levels and Memory Performance in the Suruí Indigenous from the Brazilian Amazon
Source: Int J Environ Res Public Health. 2026 Jun 12;23(6):793. doi: 10.3390/ijerph23060793 (PMC13299861; doi:10.3390/ijerph23060793)
Supplement: Supplementary file 1 [file ijerph-23-00793-s001.zip › Supplementary Table S2.pdf]

Supplementary Table S2. Linear regression models between neurological evaluation outcomes and mercury exposure levels among the 26 participants with Hg levels ≥ 2.0μg/g, Sete de Setembro Indigenous Territory, Rondônia, Amazon, Brazil, 2023.

Signs	n (%) (n=26)	Multivariable analysis[a]			
		Estimate (β)	Standard Error	95% CI	P-value
**Cognitive function**					
Verbal fluency	26 (24.1)	0.07	0.73	-1.44 ; 1.60	0.91
Memory	1 (0.9)	**3.80**	**1.38**	**0.95 ; 6.68**	**0.01**
Stick test	19 (17.8)	-0.78	0.75	-2.35 : 0.79	0.31
Cognition	46 (42.6)	0.37	0.74	-1.16 : 1.91	0.62
**Motor function**					
Muscle strength	5 (4.7)	**1.99**	**0.89**	**0.13 : 3.84**	**0.04**
Muscle rigidity	3 (2.8)	1.09	1.33	-1.67 : 3.86	0.42
Bradykinesia	3 (3.1)	<0.01	1.24	-2.63 : 2.63	0.99
Deep tendon reflexes	25 (24.0)	-0.70	0.79	-2.34 : 0.94	0.39
**Somatosensory function**					
Tactile sensitivity	5 (4.9)	-1.15	0.84	-2.90 : 0.61	0.19
Deep sensitivity	15 (13.8)	-0.93	0.68	-2.34 : 0.48	0.18
Thermal sensitivity	19 (17.4)	-1.29	0.63	-2.59 : 0.01	0.05
Nociception	17 (15.6)	-1.05	0.77	-2.65 : 0.54	0.18

[a]Adjusted for sex and age. All individuals were fish consumers
