# Supplementary material for: Influence of the GSTP1 rs1695 Polymorphism on Mercury Levels and Memory Performance in the Suruí Indigenous from the Brazilian Amazon
Source: Int J Environ Res Public Health. 2026 Jun 12;23(6):793. doi: 10.3390/ijerph23060793 (PMC13299861; doi:10.3390/ijerph23060793)
Supplement: Supplementary file 1 [file ijerph-23-00793-s001.zip › Supplementary Table S3.pdf]

Supplementary Table S3. Linear regression models between the *GSTP1* SNP and mercury exposure levels among the 26 participants with Hg levels ≥ 2.0μg/g, Sete de Setembro Indigenous Territory, Rondônia, Amazon, Brazil, 2023.

GSTP1 rs1695 A>G	Median (IQR)	Multivariable analysis[a]			
		Estimate (β)	Standard Error	95% CI	P-value
AA	2.50 (0.87)				
AG	2.67 (0.83)	-0.14	0.62	-1.43 : 1.13	0.81
GG	6.21 (0.09)	**3.05**	**1.05**	**0.86 : 5.23**	**<0.01**
AA + AG	2.57 (1.39)				
GG	6.21 (0.09)	**3.08**	**1.01**	**0.98 : 5.19**	**<0.01**
A	1.13 (0.94)				
G	0.62 (1.35)	0.98	0.49	-0.01 : 1.97	0.05

[a]Adjusted for sex and age. All individuals were fish consumers;
